# Caregiver-reported barriers to engagement in a paediatric fresh fruit and vegetable prescription programme

**DOI:** 10.1017/jns.2024.33

**Published:** 2024-09-18

**Authors:** Amy Saxe-Custack, Sarah Egan, Bridget Farmer, Kimberly Pulka, Anna Sampson

**Affiliations:** 1 Charles Stewart Mott Department of Public Health, Michigan State University–Hurley Children’s Hospital Pediatric Public Health Initiative, Flint, MI, USA; 2 Michigan State University College of Osteopathic Medicine, East Lansing, MI, USA

**Keywords:** Children, Farmers’ market, Food access, Food security, Fruits and vegetables, Nutrition incentives, Prescription

## Abstract

Paediatric fruit and vegetable prescription programmes hold promise in improving food security and dietary patterns among youth. However, programme success is largely dependent upon caregiver and family engagement. The current study sought to gain a better understanding of environmental barriers to engagement in a paediatric fruit and vegetable prescription programme in one low-income, urban community (Flint, Michigan, USA). Following the implementation of a paediatric fruit and vegetable prescription programme, researchers conducted thirty-two semi-structured interviews with caregivers. Researchers explored caregivers’ understanding of the fruit and vegetable prescription programme, barriers to programme engagement, and recommendations for improvement. Telephone interviews were transcribed for textual analysis. Researchers used thematic analysis to examine qualitative data, determine patterns across transcripts, and develop emerging themes. Researchers concluded interviews when data saturation was reached. The majority of participants were female (94%), African American (66%), and residents of Flint (72%). Five recurrent themes emerged: (1) nutrition security; (2) prescription distribution; (3) prescription redemption; (4) educational supports; and (5) programme modifications. Although caregivers indicated that the prescription programme addressed household food insecurity, environmental barriers to engagement were apparent. Caregivers provided suggestions, such as partnering with large grocery stores and developing digital prescriptions, to address programme engagement challenges. Fundamental to the success of fruit and vegetable prescription programmes is the understanding of barriers to engagement from the perspective of participants. This study explores challenges with one paediatric fruit and vegetable prescription programme and provides actionable solutions, from the viewpoint of caregivers, to address these challenges.

## Introduction

Nutrients in fruits and vegetables are important for appropriate growth and development^(1–8)^ as well as prevention of diet-related chronic health conditions.^(9–12)^ Unfortunately, most children and adolescents fall short of meeting dietary recommendations for fruits and vegetables.^(13–16)^ Because childhood represents a crucial period of growth when individuals establish enduring dietary habits,^(17–19)^ it is imperative that youth have easy access to fresh, nutrient-dense foods, such as fruits and vegetables.

Fruit and vegetable prescription programmes (FVPPs) are one strategy to increase access to fruits and vegetables for young patients.^(20–24)^ These programmes vary widely in scope and procedures; however, fruit and vegetable prescriptions are often written by primary care providers, distributed to patients, and exchanged for fresh produce at retailers, such as farmers’ markets, mobile markets, and grocery or food stores.

In addition to supporting food equity, paediatric FVPPs hold promise as both a primary and secondary prevention strategy.^(25)^ Preliminary research has shown that participation in paediatric FVPPs is associated with improvements in food security,^(21,24)^ food shopping,^(26)^ and youth dietary intake.^(21,27–30)^ Thus far, however, there is a shortage of literature that explores environmental barriers to engagement in these programmes.^(23)^ Because the effectiveness of paediatric FVPPs is largely dependent upon programme engagement among caregivers and their families, an investigation of barriers to participation is crucial. Moreover, as research continues to explore the short- and long-term implications of various models of paediatric FVPPs, qualitative research must simultaneously consider the limitations of differing programme designs.

In February 2016, Hurley Children’s Center, a large paediatric clinic in Flint, Michigan, partnered with a local farmers’ market to create Michigan’s first fruit and vegetable prescription programme solely for children.^(26,31)^ The programme provided one $15 prescription for fresh produce to every child, regardless of health status or income, at every office visit. Participants exchanged prescriptions for fresh produce at the Flint Farmers’ Market or at Flint Fresh, a local mobile market and food hub. In August 2018, this identical programme was introduced at a second paediatric clinic in Flint as part of a study to examine replicability and preliminary effectiveness.^(21,27)^ The current study sought to qualitatively explore caregiver understanding of this paediatric FVPP, barriers to programme engagement, and recommendations for improvement.

## Experimental methods

### Study setting and population

Flint, Michigan has approximately 100,000 residents and is home to General Motors. When the American automobile industry declined, Flint fell into an extreme recession.^(32)^ In addition to a child poverty rate that is approximately 50%,^(33)^ youth in Flint continue to struggle in the aftermath of a city-wide lead-contaminated water public health crisis.^(34)^ Additionally, Flint lacks nutrition resources and healthy food options. Poor quality fresh foods in local food stores limit access to nutrient-rich items, such as fruits and vegetables,^(31,35,36)^ and grocery store options in the city are scarce.^(37)^

### Paediatric fruit and vegetable prescription programme (FVPP)

Hurley Children’s Center, a residency-training paediatric clinic co-located within a farmers’ market, introduced the first paediatric fresh fruit and vegetable prescription programme (FVPP) in Michigan in February 2016. This FVPP was established to actively address enduring challenges with accessing and purchasing fresh produce among youth and families living in Flint. The FVPP was thoughtfully designed to easily integrate into the busy paediatric office while highlighting the importance of daily fruit and vegetable consumption. Prescriptions were added to the clinic’s electronic medical record (EMR) system and retained in patient records. Paediatricians ordered produce prescriptions using the EMR system and distributed the printed prescriptions to all paediatric patients at every office visit. The EMR generated monthly prescription distribution reports, which were used to track clinic-wide distribution rates.

The FVPP originally introduced at Hurley Children’s Center^(26,31)^ was expanded to one private-practice paediatric clinic in Flint in August 2018. This second clinic, Akpinar Children’s Clinic, provides care to 3000 patients, most of whom live in Flint and receive public health insurance. Identical to the original programme, the paediatrician ordered $15 fruit and vegetable prescriptions using the EMR system and gave the prescriptions to all patients during office visits. FVPP vendors included Flint Farmers’ Market, a year-round market located in downtown Flint, open Tuesday, Thursday, and Saturday from 9 AM until 5 PM, and Flint Fresh, a mobile market and food hub, that offered free delivery of participant-selected fresh produce boxes. Prescriptions were treated as vouchers that could only be exchanged for fresh fruits and vegetables and were valid for 90 days. Participants could not divide their prescription between multiple farmers’ market vendors, and any remaining balance from a transaction totalling less than $15 was lost.

From August 2018 through March 2019, 365 caregiver–child dyads at Akpinar Children’s Clinic provided written consent and assent to join a study assessing the feasibility of the FVPP,^(21,27)^ including follow-up interviews to assess programme experiences.

### Approach and theoretical framework

The study design and approach followed the theoretical framework of Bandura’s Social Cognitive Theory (SCT). SCT explains behaviour through a 3-stage model connecting personal factors, environmental factors, and behaviour.^(38)^ Since children’s nutrition choices are typically guided by their caregivers,^(39,40)^ a qualitative investigation of environmental factors that prevented caregivers from fully engaging in the programme with their children was of particular importance.

### Participants and data collection

Researchers collected data via semi-structured telephone interviews between December 2022 and March 2023. Distribution data (EMR reports) and redemption data (redeemed paper prescriptions) were used to identify children who enrolled in the original study with their caregiver but failed to redeem fresh produce prescriptions during the approximate four-year study period. To assess barriers to engagement in the FVPP as well as caregiver experiences with the programme, an open-ended interview format was created.

Caregivers were eligible to participate in interviews if: (1) they enrolled in the original study and completed baseline surveys; (2) their enrolled child had received at least one fruit and vegetable prescription; (3) their enrolled child had not redeemed any $15 fruit and vegetable prescriptions during the study period; and (4) their enrolled child was an active patient at Akpinar Children’s Clinic. A total of 98 caregivers met eligibility requirements. Researchers attempted to contact all 98 caregivers but were challenged with non-working or disconnected telephones. After 32 interviews were completed, researchers concluded that no new concepts were arising and that data saturation had been reached. Participants received one $50 electronic gift card after completing the interviews.

The open-ended interview format was used to detect caregiver understanding of the FVPP, barriers to programme engagement, and recommendations for improvement. One-on-one interviews were led by three research team members trained in qualitative research methods. Questions such as *In your own words, can you explain the fruit and vegetable prescription program and how it works* invited conversation regarding caregiver understanding of the programme and redemption procedures, while more involved questions such as *What prevented you from redeeming all of your prescriptions* and *Do you have specific thoughts or ideas to change the program to address the barriers you have experienced redeeming your prescriptions* probed about specific challenges related to programme engagement. Researchers gathered additional information using an interview guide informed by previous literature,^(31,41–44)^ research questions, and experiences with the subject matter and population.

### Data analysis

All caregiver interviews were audio recorded and transcribed verbatim for textual data analysis. Researchers examined data by following a coding process informed by thematic analysis. During initial coding, four researchers individually analysed transcripts and identified notable patterns for thematic purposes. Researchers then met to collapse similar themes and determine final emerging themes. Lastly, three researchers selected illustrative direct quotes to represent the final themes and sub-themes.

This study was conducted according to the guidelines laid down in the Declaration of Helsinki and all procedures involving human subjects were approved by the Michigan State University Institutional Review Board – Study 00000666. Written informed consent was obtained from all subjects.

## Results

A total of 32 interviews with caregivers were completed. Interview participants (mean age, 40.9 ± 9.4 years) were primarily female (94%), African American (66%), and residents of Flint (72%) (Table 1). These demographics are representative of the full sample of 365 caregivers who enrolled in the larger study (mean age 39.7 ± 9.9 years), most of whom were female (91%), African American (66%), and residents of Flint (72%).

**Table 1.** Characteristics of caregivers who completed interviews

	Caregiver characteristics	Frequency *n*	%
Age ± SD (Range)	40.88 ± 9.35 years (29–78 years)		
Gender	Female	30	94
	Male	2	6
Race	African American	21	66
	Caucasian	8	25
	Hispanic	1	3
	Native American/American Indian	1	3
	Other	1	3
Residence	Flint	23	72
	Non-Flint	9	28
Education	Some high school/High school	7	22
	Trade school	2	6
	Associate’s degree	12	38
	Bachelor’s degree	8	25
	Graduate or professional degree	3	9

Important to research findings was the general feedback from most caregivers that their household had redeemed at least one prescription during the study period. Although study records accurately tracked and recorded redemption rates for children enrolled in the study, records failed to capture prescription redemption for siblings living in the same household but not enrolled in the study. Only eight of the 32 caregivers interviewed (25%) acknowledged that no child in their household had ever redeemed a produce prescription. We present the following recurrent themes and associated findings, each centred around caregiver experiences with the FVPP: (1) nutrition security; (2) prescription distribution; (3) prescription redemption; (4) educational supports; (5) programme modifications. These themes can be found in Table 2, corresponding with the associated sub-themes.

**Table 2.** Illustrative quotes collected from caregivers of children enrolled in a produce prescription programme

Theme	Sub-theme	Illustrative quote
1. Nutrition Security	1.1 Financial Support	“My sister doesn’t get any type of help from the state. So, she pays cash for everything. By you guys having this program, she gets some food and she loves going and spending those coupons on it.” (Participant 176, African American Female, Age 43)
		“Right now [prescriptions] would be a huge help because my husband hasn’t worked in a year and a half. So, I’m working seven days a week, two jobs, trying to keep food on the table, roof over our heads, electricity, all that stuff. I think [the prescription program is] fantastic.” (Participant 267, Caucasian Female, Age 56)
		“I think it’s great that they are doing it and helping people get fresh fruits and vegetables, especially people that can’t afford it. They are expensive. It’s more expensive to eat healthy than it is to eat unhealthy.” (Participant 303, Caucasian Female, Age 36)
	1.2 Nutrition and Health	“I think it’s an amazing program. It gives people an incentive. I work in the medical field, and not everyone takes their child to visit the doctor. A lot of times you see, like urgent cares or emergency rooms being overused for things they don’t need to be used for. I think giving them an incentive like [produce prescriptions] brings people to bring their child in for a well visit.” (Participant 198, Caucasian Female, Age 34)
		“I think it’s a great program because it helps parents not only with their grocery bills, but it makes sure that their children are receiving fruits and vegetables that are vital to good health, and to help them grow.” (Participant 267, Caucasian Female, Age 56)
		“I think [the prescription program is] a good way to introduce the importance of fruits and vegetables to the community. It is introducing it to children as young as infants, and parents to ensure that you know the importance of it.” (Participant 302, Native American/American Indian Female, Age 33)
	1.3 Child Engagement	“I think the best [part of the] program is that the kids are involved! When I go with my daughter, she picks out the fruits that she wants or she likes to eat. It’s kind of a bonding thing for us.” (Participant 085, African American Female, Age 53)
		“When we see [pediatrician] and receive the prescription, [my son] knows that’s for him to go pick out his own fruits and vegetables. I leave that on him, to pick out the ones he wants.” (Participant 198, Caucasian Female, Age 34)
		“They [kids] are definitely helping because if I pick it, they don’t want it.” (Participant 302, Native American/American Indian Female, Age 33)
2. Prescription Distribution	2.1 Procedures	“I don’t think it was every time I went. Like for a check-up or something… I thought [prescriptions] were given if [the clinic] wanted to give them to us. I didn’t know it was for every time you went in there.” (Participant 015, African American Female, Age 43)
		“I didn’t know if [pediatrician] was still doing it or not. That’s why I was asking my friends because I didn’t get one [prescription] last time. I thought maybe the program ended.” (Participant 090, Caucasian Female, Age 35)
		“[My son] just came to get a meningitis shot and a physical last week. He didn’t get [a prescription].” (Participant 153, African American Female, Age 50)
	2.2 Program Understanding	“I’m not sure how it works. I know they give you a prescription, some for fruits and vegetables, but I didn’t use it because I didn’t know how to use it.” (Participant 082, African American Female, Age 42)
		“When I’ve been grocery shopping, I’ve seen where it says you can get the fruit and vegetables, buy one get one free or something like that. Is that the same program?” (Participant 190, African American Female, Age 53)
		“I’m not all clear. It’s a guy in like a food truck. I forget what it’s called…. Sometimes I couldn’t get to the farmers’ market so Fresh Start [a different program] was the better choice.” (Participant 037, African American Female, Age 39)
	2.3 Delivery Option	“I didn’t realize that they have a delivery. I never find time to go to the famers’ market because I live on the other side of town.” (Participant 306, Caucasian Female, Age 36)
		“I didn’t know they delivered, and I never really had time to go to the farmers’ market.” (Participant 347, African American Female, Age 36)
		“I didn’t know that they delivered… a couple of months ago I heard about it but I’m like, ‘Nah they wouldn’t come all the way out here’ but because I’m in [a different city].” (Participant 290, African American Female, Age 33)
3. Prescription Redemption	3.1 Vendor Site Navigation	“It’s not labeled how much things are at the farmers’ market. I was just worried about going over the amount and not having the money on me to cover it. I just didn’t want to be in a weird position.” (Participant 090, Caucasian Female, Age 35)
		“In the beginning we did not know [where to redeem at the farmers’ market], and that’s why we weren’t using prescriptions as much.” (Participant 085, African American Female, Age 53)
		“At first it’s a little confusing trying to figure out where you go [on the Flint Fresh website] because the paperwork is completely different from the website.” (Participant 306, Caucasian Female, Age 36)
	3.2 Paper Prescriptions	“When my kids go to the doctor, and they give me paperwork, I normally take that paperwork and file it away. Then, by the time I remember about the prescriptions and pull them out, they are already expired.” (Participant 129, Caucasian Female, Age 36)
		“I lost the prescription. I’m not sure what I did with it. We had some things we had to do after the last visit, and I must have set it down somewhere. You know, out of sight, out of mind.” (Participant 198, Caucasian Female, Age 34)
		“I just forget that I have them. And if they are not in my purse or wallet, I don’t pay any attention.” (Participant 273, African American Female, Age 45)
	3.3 Farmers’ Market	“One thing was getting to the farmers’ market. I worked a lot, and it was hard. It’s hard to get to the farmers’ market on the days they are open. And when I get there, either I have forgotten about [prescriptions] or it’s so busy and so packed where I don’t have time for them to explain to me how to go about using the prescription.” (Participant 082, African American Female, Age 42)
		“The farmers’ market is only open on certain days… And a day that we may have available as a family they may not have been open. And I do recall that, once or twice, a scenario where we just couldn’t go that day.” (Participant 312, Caucasian Male, Age 39)
		“I work a lot. And, by the time I get out of work, the farmers’ market is closed.” (Participant 362, African American Female, Age 37)
	3.4 Transportation	“I have had so many car issues the last couple of years. I just can only really make it to where I need to go and nowhere else.” (Participant 129, Caucasian Female, Age 36)
		“Just living so far away… I have a vehicle and all that, but we’re a low-income family. It’s just hard to get out there and do it. We just shop locally.” (Participant 303, Caucasian Female, Age 36)
		“Now, [a barrier to redeeming prescriptions] would be a transportation issue because I totaled my car out.” (Participant 082, African American Female, Age 42)
4. Educational Supports	4.1 Culinary Programs	“Suggestions for healthy ways to get the kids to eat the vegetables. Something like that because I don’t think the struggle is people wanting to feed their kids the thing. I think it’s getting them to eat it.” (Participant 128, African American Female, Age 43)
		“A cooking class where they taught you like how to take cauliflower and make bread crust, like a crust to a pizza out of cauliflower. Healthier options… a healthy dinner where you are getting your vegetables or your fruit.” (Participant 198, Caucasian Female, Age 34)
		“I know we have COVID and stuff, but maybe once a week, a healthy cooking class?” (Participant 302, Native American/American Indian Female, Age 33)
	4.2 Recipes	“If they had pamphlets of recipes or something like that. At school they learn about a vegetable or a fruit, and they get sent home a little recipe card. My kids really enjoy those.” (Participant 129, Caucasian Female, Age 36)
		“Maybe a digital link that will give different recipes to use fruits and vegetables that are available to us.” (Participant 166, African American Female, Age 50)
		“I think a cookbook would be nice.” (Participant 273, African American Female, Age 45)
	4.3 Virtual Format	“Maybe like a Facebook Live or YouTube video. Where they pull it up at their convenience. I think it would get a lot more people watching it. ‘This is what we are going to make this week. This is what we are going to need.’ So, everything is there, right in front of them.” (Participant 267, Caucasian Female, Age 56)
		“I wouldn’t be able to take part in the cooking, unless it’s online or something… due to my work schedule.” (Participant 285, African American Female, Age 42)
		“Most of the time it’s just somebody finding the time…a lot of people aren’t showing up to certain things because COVID and stuff is still around. So virtual is definitely a good thing.” (Participant 306, Caucasian Female, Age 36)
5. Program Modifications	5.1 Prescription Format	“It would be nice if it was on a card or something. So, you can redeem everything on the card and don’t have to keep up with the [prescription] paper. Just one [card] for the whole family…When you go to the market, you just have a card that tells you, ‘You have this amount on there to buy fruits and vegetables.’” (Participant 033, African American Female, Age 30)
		“I think if there was a card, reloadable for the prescriptions, that would be a lot easier. Because everywhere, every store around, has their own cards and phone number to enter. If they had something like that, it would be easier to not lose.” (Participant 198, Caucasian Female, Age 34)
		“If [the prescription] was available on an app, we as a family would have redeemed absolutely every single one, without question.” (Participant 312, Caucasian Male, Age 39)
	5.2 Redemption Sites	“It would be nice if [the prescription] was good at local grocery stores as well… the main ones that’s in my area would be very helpful.” (Participant 033, African American Female, Age 30)
		“Could it be something that could expand to maybe Meijers or Krogers or I don’t know Walmart? I don’t know if it could be, but it’s a suggestion.” (Participant 085, African American Female, Age 53)
		“Some people live in those neighborhoods, so it’s more convenient for them to walk down to the store and get those items if they can’t make it to the farmers’ market.” (Participant 166, African American Female, Age 50)
	5.3 Expiration Date	“If maybe they took the expiration date away…I think that expiration date thing would help a lot.” (Participant 037, African American Female, Age 39)
		“I wish they would let you go like maybe a year or so because I always file [prescriptions] away and then forget about it. By the time I find it or come across again, they have already expired.” (Participant 129, Caucasian Female, Age 36)
		“They should set a reminder like, ‘Hey, [the prescription is] about to expire.’ Cause us as parents, we forget things. We get busy with our kids and forget things. So, a text reminder, like ‘Hey, your benefits will expire by such and such date.’ I think that would be helpful.” (Participant 290, African American Female, Age 33)

### Nutrition security

Nutrition security is defined as ‘consistent access, availability, and affordability of foods and beverages that promote well-being, prevent disease, and, if needed, treat disease, particularly among racial/ethnic minority populations, lower income populations, and rural and remote populations’.^(45)^ Most caregivers indicated that they had redeemed at least one produce prescription with a child in their household during the study period. These caregivers perceived the FVPP to have meaningful impacts on nutrition security.

A majority of caregivers expressed gratitude for the FVPP as a financial support for their families (Table 2, sub-theme 1.1). Many noted growing concerns regarding the cost of healthy foods, particularly fresh fruits and vegetables, and appreciated the FVPP for alleviating that burden. Some further noted the importance of paediatric office visits, accompanied by produce prescriptions, in directly combating the growing costs of fresh foods.

*Sometimes it can be really expensive to get healthy fruits and vegetables. And it’s $15 worth of fruits and vegetables that I don’t have to pay out of pocket… It helps the pockets of low-income families.* (Participant 180, African American Female, Age 43)

*Since COVID, vegetables are going up [in price]. I don’t get food stamps, so I took the kids to their appointment today. At least I know I’ll get some kind of help for vegetables, fruits and vegetables, by coming in [to the doctor].* (Participant 285, African American Female, Age 42)

Many caregivers recognised the value of the prescription programme in supporting the nutrition and health of their children (Table 2, sub-theme 1.2). Some noted their appreciation for paediatricians who offered the programme, rather than medicine or pills, to actively encourage disease prevention and healthy eating among young patients. Many felt the programme served as an incentive for families to bring their children to the paediatrician.

*I think it’s really great that the doctors care enough about our health to want to give us free fruits and vegetables and teach us about nutritional meals… I think that’s definitely the best part, to go to the doctor and walk out with a prescription that’s not a pill. It’s like they are actually wanting you to eat healthy.* (Participant 129, Caucasian Female, Age 36)

Many caregivers talked about child engagement in the FVPP, particularly with regard to produce selection at the farmers’ market (Table 2, sub-theme 1.3). Some further described how they planned trips to the farmers’ market to use their prescriptions as an opportunity to educate and bond with their children.

*The kids thoroughly enjoyed, and I do mean thoroughly enjoyed, going to the Flint Farmers’ Market giving the vendors their [prescriptions], and picking out their own vegetables.* (Participant 312, Caucasian Male, Age 39)

### Prescription distribution

Caregivers were asked to explain the FVPP in their own words. Although the majority of caregivers had a general understanding of the programme, key components were consistently unclear among interview participants. Most confusion occurred at the point of distribution (i.e. receipt of produce prescriptions at the paediatric clinic).

In contrast with programme distribution procedures that specified every child should receive one $15 prescription at each office visit, most caregivers indicated that prescriptions were not given during every office visit (Table 2, sub-theme 2.1). Some believed the prescriptions were reserved only for annual well-visits, while others simply explained that prescription distribution was inconsistent across office visits.

*If I do a walk-in visit, I don’t get nothing for that. It’s only a scheduled appointment that I ever got [prescriptions].* (Participant 005, African American Female, Age 34)

*I received it usually when we do their yearly check-ups. Any other time I was there, it would be an emergency call or something like that. I would receive them [only] when we do the check-up yearly.* (Participant 082, African American Female, Age 42)

Nearly every caregiver said that an introduction to the FVPP was provided at the paediatric office when their child received their first prescription. In spite of this programme introduction, many shared that their programme understanding was inadequate (Table 2, sub-theme 2.2). Some confused the FVPP with other food assistance and nutrition incentive programmes. Several admitted that their poor understanding of the FVPP influenced their decision not to redeem prescriptions.

*I was trying to take everything in on how to do it. Honestly, I didn’t fully understand how to [use prescriptions].* (Participant 036, Hispanic Female, Age 44)

*I didn’t redeem them because of the fact that I didn’t understand that they were for fruits and vegetables. The fresh things at the market.* (Participant 085, African American Female, Age 53)

When describing the prescription programme in their own words, most caregivers talked only about the local farmers’ market as the redemption site for prescriptions. A majority of caregivers were entirely unaware of the free produce delivery option through Flint Fresh (Table 2, sub-theme 2.3). Some, who were aware that Flint Fresh was a vendor for the FVPP, lacked general knowledge of this option, such as delivery procedures, radius, or associated costs.

*I didn’t even know they had delivery.* (Participant 166, African American Female, Age 50)

*I was afraid with the delivery service that I was going to be charged. And money is tight, it’s still tight, but I think the delivery service is a really good idea.* (Participant 267, Caucasian Female, Age 56)

### Prescription redemption

When asked to explain specific barriers to engagement in the FVPP, most caregivers talked extensively about prescription redemption. Some were challenged when navigating redemption sites, both in-person at the farmers’ market and online through Flint Fresh. Caregivers also discussed conflicts related to work hours and farmers’ market hours, challenges with transportation, and management of paper prescriptions. Each of these, alone or in combination, acted as barriers to engagement in the FVPP.

When arriving at the farmers’ market or placing an order on the Flint Fresh website, many caregivers shared their frustration with vendor site navigation (Table 2, sub-theme 3.1). Some were unsure which farmers’ market vendors accepted prescriptions; while others were unclear regarding specifics of redemption, such as exceeding prescription value or splitting the prescription value between vendors. Some caregivers, who attempted to redeem prescriptions through the Flint Fresh website, described technical challenges.

*When I got to the farmers’ market and walked in, I didn’t know where to go. So, that’s where I got lost.* (Participant 290, African American Female, Age 33)

*When I went on the website to try to order [a produce box], for some odd reason it wouldn’t let me continue. I picked all my stuff, but it wouldn’t let me submit it.* (Participant 176, African American Female, Age 43)

Although every caregiver expressed genuine appreciation for the prescription programme, many shared continual difficulties related to lost or forgotten paper prescriptions (Table 2, sub-theme 3.2). Some further indicated that when found, prescriptions were very often expired (beyond the 90-day expiration date). A majority of caregivers indicated that the format of prescriptions, small pieces of prescription paper, made tracking and management of the incentive difficult.

*I lose them often because it’s such a small piece of paper. I just misplace them. When I do find them again, they are expired. So, that’s a big issue.* (Participant 090, Caucasian Female, Age 35)

Many caregivers discussed the exceptional quality of produce and generosity of farmers’ market vendors. However, some commented on limitations of the farmers’ market as the primary produce prescription programme redemption site (Table 2, sub-theme 3.3). Most indicated that the limited hours of operation of the farmers’ market (Tuesday, Thursday, and Saturday from 9:00 AM to 5:00 PM) conflicted with work or family schedules. Some further shared that distance to the farmers’ market or anticipated crowds discouraged prescription redemption there.

*It is kind of hard, especially working nine to five… Really the only day I have available, if I’m available that day, is a Saturday.* (Participant 180, African American Female, Age 43)

Flint Farmers’ Market is located in downtown Flint. It is easy to access through public transportation, specifically through bus routes. Still, many caregivers discussed challenges related to reliable transportation to the farmers’ market (Table 2, sub-theme 3.4). Some shared problems specifically related to functioning cars, while others felt unsafe accessing public transportation.

*I think over the years I’ve probably redeemed them [prescriptions] two or three times only because of lack of transportation.* (Participant 166, African American Female, Age 50)

*There was one point in time when there was a transportation issue. Even though I did have access to the bus, it wasn’t a safe area to get on the bus. Even though I had access to the bus line, where the bus line was, it wasn’t safe to get on.* (Participant 302, Native American/American Indian Female, Age 33)

### Educational supports

When asked to describe the type of education that caregivers felt would benefit their families, many indicated that they would like cooking classes or recipes to be offered alongside prescriptions. Some further suggested that educational programmes or sessions should be offered in a virtual format.

Most caregivers requested that culinary programmes be offered with the FVPP (Table 2, sub-theme 4.1). Some caregivers shared struggles encouraging their children to eat more fruits and vegetables and desired a class that would address this challenge. Others believed a youth-focused cooking class would be beneficial.

*I have a 15-year-old daughter, and she absolutely would love some [culinary] education if it was presented to her. “Hey, go to the farmers’ market, go buy these things and then you can use them to cook XYZ”.* (Participant 312, Caucasian Male, Age 39)

Nearly all caregivers requested that recipes accompany produce prescriptions (Table 2, sub-theme 4.2). Some felt recipes should be distributed with prescriptions; while others suggested fruit and vegetable recipes be provided through a website, regular emails, or farmers’ market kiosk.

*Recipes would be awesome… Email or at the doctor. Just have the attendant print [recipes] off. Just hand recipes to you while you are at the doctor’s office. Just like [pediatrician] does with the prescription.* (Participant 015, African American Female, Age 43)

*A website or recipes you could download. For instance, when you get the prescription, maybe there would be a website for quick meals or quick recipes. Or when we go to the farmers’ market, they give out free recipes or something.* (Participant 085, African American Female, Age 53)

Many caregivers suggested that if programme education, including nutrition or culinary instruction, were to be offered with the prescription programme, sessions should be presented in a virtual format (Table 2, sub-theme 4.3). Some noted busy schedules and competing priorities, while others pointed to struggles with transportation. Most felt that offering education virtually would address these barriers.

*It’s kind of inconvenient sitting in a class when you’ve got so much to do. I believe something on a QR code or app. So, [participants] can just go on and look it up and maybe even a video to help people and show them what to do.* (Participant 033, African American Female, Age 30)

*A class or something through Zoom. So that even if a person doesn’t have transportation, most people have cell phones, even the free government phones. So, they would be able to go to a Zoom meeting for the purpose of learning how this program works and things about nutrition.* (Participant 190, African American Female, Age 53)

### Programme modifications

Throughout the interviews, caregivers were candid regarding modifications that should be made to the current programme to improve engagement. Most interview participants had redeemed at least one produce prescription, and many felt changes could be made to improve the programme experience for families.

The majority of caregivers felt strongly that the paper prescription format needed to be changed (Table 2, sub-theme 5.1). Caregivers offered thoughtful solutions to address the challenges with paper prescriptions. Most clearly indicated a desire for prescriptions to be available through a card or an application (i.e. app) for the entire household. Some further suggested that prescription cards or apps should automatically reload for members of the household following clinic appointments.

*An app that’s under an umbrella of all my kids versus individually and me having one for each kid at random times… Just having multiple kids and sending random papers [prescriptions] attached to check-out stuff. I think an app, where I know it’s going to be there, already loaded and ready to go. I think that would be more helpful.* (Participant 005, African American Female, Age 34)

*A card instead of paper. Because paper is something that can really easily get misplaced. But a card, you can put it inside of your wallet and you’ll be able to see it and say, “Oh yeah, let me go use this.” Even a reusable card that once you go in [to the pediatrician’s office], they just refill it for you.* (Participant 015, African American Female, Age 43)

Although most caregivers expressed fondness for the farmers’ market, many felt the programme should expand to include more redemption sites (Table 2, sub-theme 5.2). Specifically, caregivers felt that expansion to full-service grocery stores would increase programme engagement due to their broad selection of food items that would allow caregivers to redeem prescriptions while grocery shopping. Some also mentioned that grocery stores were closer to their homes and more convenient to shop at than the farmers’ market.

*[Prescriptions] are only good at the farmers’ market, and I don’t always have the time to go to the farmers’ market. I do a lot of my shopping at [grocery stores], and I can go straight there. I’m not big on going to multiple stores… I like to just go to where I am going and go back home. I have a lot of children, so I don’t really have a lot of time to stop at the farmers’ market as well as other grocery stores.* (Participant 033, African American Female, Age 30)

Produce prescriptions expired 90 days from the date of distribution. Many caregivers expressed frustration with these expiration dates (Table 2, sub-theme 5.3). Some asked that the expiration dates be removed from the prescriptions entirely. Others requested that reminder texts or emails be sent to caregivers when prescriptions were nearing their expiration date.

*Just a small text message or an email… “Don’t forget to use your coupon by this date.” Like when the doctor reminds you that you have a doctor’s appointment. Because sometimes, as parents, we get overwhelmed and sometimes forget. So, a reminder would definitely help.* (Participant 015, African American Female, Age 43)

*I don’t know why they have an expiration date. I did go one time, and I was excited to know that I had one of those prescriptions. But it was expired. So, maybe if they didn’t have the expiration dates.* (Participant 036, Hispanic Female, Age 44)

## Discussion

The current study is among the first to explore and describe caregiver-reported barriers to engagement in a paediatric FVPP. Most caregivers reported that they had redeemed at least one produce prescription with a child in their household and maintained deep gratitude for the programme that served as both a financial and nutritional support for their children. However, many were also forthcoming about the need for programme improvements to increase overall engagement. Central to our findings was a lack of awareness regarding specific features of the programme, such as prescription distribution schedules, vendor details, and redemption rules. Many caregivers further indicated that this lack of clarity influenced their decision not to redeem prescriptions. Additional environmental barriers to redemption included transportation challenges, management of small pieces of prescription paper, and limited farmers’ market selection and hours. Caregivers offered practical suggestions, such as digital prescriptions and partnerships with full-service grocery stores, to address many of the challenges identified during interviews.

Similar to previous qualitative work, caregivers expressed a genuine appreciation for the FVPP, recognising that healthcare providers offered this programme out of concern for the health and well-being of their young patients.^(31)^ Some even indicated that the programme served as a motivation for attending regular office visits or as a deterrent from utilising urgent or emergency care services. Although research examining health care utilisation among participants in a paediatric FVPP is unavailable, recent findings suggest that food prescription programmes may reduce emergency department utilisation among adults.^(46)^ Given the importance of regular paediatric visits for immunizations and preventative care, research focused on the influence of paediatric FVPP on health care utilisation is needed. Additionally, feedback from caregivers also signalled a recognition of the impact of the FVPP on nutrition security. Many talked extensively about the growing cost of fresh, healthy foods, with some suggesting that it was more cost-effective to purchase convenience or packaged foods. The paediatric FVPP was viewed as a means for caregivers to provide fresh fruits and vegetables to their households, even as the cost of these items was increasing. This finding is consistent with previous research that indicates paediatric FVPPs have a positive influence on nutrition and food security among youth and their families.^(21,24,31)^

Similar to research with adults,^(47)^ many caregivers shared a lack of understanding of the paediatric FVPP at the point of distribution (receipt of prescriptions). This finding illustrates the need for a consistent programme education plan and supporting materials at partnering clinics. Previous research has suggested that, in order for FVPPs to run effectively, a paid staff member should be responsible for coordinating the programme at clinics and managing education, referrals, and challenges.^(48)^ Given the numerous demands on paediatric clinics and staff, it may be unreasonable to expect consistent in-person education regarding a paediatric FVPP without one dedicated staff member on-site. Although posters and pamphlets, which described vendor addresses, hours of operation, and redemption procedures, were available at the partnering clinic, many caregivers remained unclear about participating vendors and redemption procedures. Some admitted that this lack of programme understanding negatively influenced their engagement in the programme.

Studies among adults who participated in fruit and vegetable incentive programmes have highlighted several barriers to engagement, including insufficient redemption/vendor sites^(49–51)^ and lack of transportation.^(47,52)^ Caregivers in the current study reported similar challenges that prevented them from engaging in the paediatric FVPP with their children. The FVPP partnered with Flint Fresh (Flint Fresh | Fresh Vegetable & Fruit Delivery in Flint) in January 2018 to preserve produce selection capacity while directly addressing transportation barriers among participants. Flint Fresh is a food aggregation space for local farmers and provides free delivery of participant-selected $15 and $30 fresh produce boxes. Unfortunately, few participants were aware of this option which resulted in underutilisation of prescriptions at this redemption site. Additionally, some shared confusion when attempting to navigate the farmers’ market and Flint Fresh website, highlighting a need for programme education at vendor sites and clinics. Future research will explore the impact of a community health worker, who specifically addresses challenges with programme education at both clinic and vendor sites, in improving engagement in the paediatric FVPP.

In February 2024, one major chain grocery store in Michigan partnered with the FVPP to accept fruit and vegetable prescriptions at seven of its stores in Flint and surrounding areas. Patients at Akpinar Children’s Clinic may now select Flint Farmers’ Market, Flint Fresh or Meijer grocery stores to redeem their prescriptions. Printed prescriptions may be redeemed at the pharmacy counter at Meijer stores in exchange for $15 fresh fruit and vegetable vouchers. Early results suggest this option has greatly improved engagement in the FVPP, and caregivers have expressed consistently positive experiences with the additional redemption site. The partnership was in direct response to caregiver feedback regarding the need for inclusion of at least one major chain grocery store where families may redeem prescriptions while shopping for other food items for their families.

Unlike most prescription programmes, participation in nutrition education activities was not required by the paediatric FVPP in the current study. Most caregivers indicated a desire for recipes or cooking classes to accompany prescriptions. Some requested cooking classes centred around children to teach culinary skills together with nutrition education. Others felt they would benefit from a culinary programme that focused on the fruits and vegetables that were purchased with prescriptions. Findings are consistent with earlier studies among adult and youth participants in FVPPs^(31,49)^ and speak to a growing need for culinary support programmes. In direct response to early feedback from FVPP participants regarding a need for culinary programmes for youth,^(31)^ Flint Kids Cook was developed in 2017.^(53)^ Flint Kids Cook is a free six-week cooking and nutrition programme for youth (aged 8–18 years) taught by a professional chef and registered dietitian in a farmers’ market kitchen. Results suggest that participation in Flint Kids Cook is associated with significant improvements in cooking attitudes, cooking self-efficacy, and health-related quality of life of participating youth.^(54)^ A virtual version of the class, Flint Families Cook, launched in 2021 with results suggesting programme participation was associated with improvements in cooking self-efficacy, health-related quality of life, and dietary behaviours.^(55,56)^ Over 500 youth have graduated from Flint Kids Cook with nearly 300 children waiting to participate. All paediatric clinics offering the FVPP advertise Flint Kids Cook through posters and brochures in waiting areas and patient rooms.

Finally, caregivers provided tangible recommendations to improve overall engagement in the paediatric FVPP. Some suggestions, including text or email reminders when prescriptions were set to expire, would be relatively easy to implement and have been reported in earlier qualitative studies with adults.^(49)^ Other suggestions, including partnerships with large grocery store chains and digitising paper prescriptions, would require significant changes that may be costly to implement and require dedicated staff and procedural changes. Unfortunately, sustainable funding sources that allow produce prescription programmes to invest in these more costly initiatives to improve programme engagement are limited. Given the potential of paediatric FVPPs as a strategy to address inequities in food access in a broadly scalable manner,^(25)^ substantial investment in continued programme improvements is warranted.

This current study has limitations. It was small and limited to one urban community in Michigan. Results may not be generalisable. As previously mentioned, because only one child and one caregiver enrolled into the original study, researchers were unable to accurately quantify the total number of prescriptions received and redeemed by each household. Finally, it is possible that views of the programme and challenges with redemption differed among participants researchers did not reach for interviews. However, participants were candid about their programme experiences and barriers to redemption. Each offered important feedback to improve overall engagement.

## Conclusions

The potential impact of fruit and vegetable prescription programmes focused on paediatric patients is substantial. In addition to recent studies indicating a positive influence on caregiver- and child-reported food security,^(21,24)^ food shopping,^(26)^ and dietary behaviours of children,^(21,27–30)^ these programmes offer notable benefits to all household members who consume produce purchased with prescriptions. Crucial to the success of these programmes, however, is the understanding of barriers to engagement among programme participants and their families. The current study elucidates challenges with one paediatric FVPP and provides actionable solutions, from the viewpoint of caregivers, to address these challenges. Future research will investigate whether and how expansion to full-service grocery stores and development of digital prescriptions impact programme engagement.

## Abbreviations

**FVPP:** Fruit and vegetable prescription programme; **EMR:** Electronic medical record; **SCT:** Social Cognitive Theory.
